# Changes in dairy product consumption and subsequent type 2 diabetes among individuals with prediabetes: Tehran Lipid and Glucose Study

**DOI:** 10.1186/s12937-021-00745-x

**Published:** 2021-10-29

**Authors:** Emad Yuzbashian, Golaleh Asghari, Parvin Mirmiran, Catherine B. Chan, Fereidoun Azizi

**Affiliations:** 1 Department of Agricultural, Food and Nutritional Science, University of Alberta, Edmonton, Alberta Canada; 2 Nutrition and Endocrine Research Center, Research Institute for Endocrine Sciences, Shahid Beheshti University of Medical Sciences, Tehran, Iran; 3 Department of Clinical Nutrition and Dietetics, Faculty of Nutrition Sciences and Food Technology, National Nutrition and Food Technology Research Institute, Shahid Beheshti University of Medical Sciences, Tehran, Iran; 4 Department of Physiology, University of Alberta, Edmonton, Alberta Canada; 5 Endocrine Research Center, Research Institute for Endocrine Sciences, Shahid Beheshti University of Medical Sciences, Tehran, I R Iran

**Keywords:** Milk, Fermented dairy, Yogurt, Cheese, Glucose homeostasis

## Abstract

**Background:**

People with prediabetes can postpone or even reverse progression to type 2 diabetes (T2D) by making dietary changes. This study aimed to examine the association of changes in consumption of total and specific types of dairy products with the subsequent risk of incident T2D among individuals with prediabetes.

**Method:**

This cohort study included 639 individuals (50% female, mean age 47.3 years) of the Tehran Lipid and Glucose Study (TLGS) who had prediabetes at baseline. We assessed 3-year changes in the consumption of dairy products using a food frequency questionnaire. Using multivariable logistic regression, odds ratios (OR) and 95% confidence intervals (CI) were calculated for the association of changes in intake of total and subtypes of dairy products during a 3-year interval with the risk of incident T2D in the subsequent 3 years.

**Results:**

After almost 9 years of follow-up, the incidence of T2D was 25.2%. Compared with individuals whose intake remained relatively stable over 3 years, those who decreased consumption of total dairy (> 0.5 servings/day) had a higher T2D risk (OR = 1.56; 95% CI: 1.02 to 2.41). Increasing low-fat dairy consumption by 0.50 serving/d was associated with a lower risk of T2D (OR = 0.56; 95% CI: 0.35 to 0.90) compared with stable consumption. Those who increased consumption of low-fat milk (OR = 0.59; 95% CI: 0.37 to 0.92) and low-fat yogurt (OR = 0.55; 95% CI: 0.33 to 0.93) had a lower risk of T2D than those who were relatively stable in their consumption. Replacing low-fat milk and yogurt with regular cheese was associated with 66 and 47% higher risk of T2D, respectively.

**Conclusion:**

In individuals with prediabetes, increasing consumption of low-fat dairy, low-fat milk, and low-fat yogurt had reduced risk of subsequent T2D. These data suggest a role of low-fat dairy products in the prevention of T2D among prediabetes patients.

**Supplementary Information:**

The online version contains supplementary material available at 10.1186/s12937-021-00745-x.

## Introduction

Prediabetes is a health condition characterized by dysregulation of glucose homeostasis that temporarily presents between the normal metabolic state and the progression to overt type 2 diabetes (T2D) [1]. Individuals with prediabetes are highly prone to developing cardiovascular disease [2] and the possibility of lifetime conversion to T2D is up to 70% [3]. The prevalence and incidence of prediabetes are rising worldwide [4, 5] and, by 2035, the International Diabetes Federation estimates a prevalence of prediabetes of 471 million people globally [6]. Diet modification is recommended as an effective strategy in patients with prediabetes to prevent or delay the onset of T2D and other complications [3, 7]; however, the literature on the association of dietary intake with incident T2D among individuals with prediabetes is limited.

Foods classified as dairy products are widely consumed in many parts of the world and supply protein and minerals necessary for human health [8]. However, dairy products also contain factors conferring health risks, including cholesterol and saturated fatty acids (SFAs), though the mixture of SFA found in dairy does not raise the cardio-metabolic risk to the same extent as other foods [9, 10]. Likely because of the interplay of various nutrients and processing characteristics, association studies of the amount and type of dairy foods with T2D risk demonstrate mixed results [11–13]. A possible mediating effect of baseline glycemic status on risk conferred by intake of various types of dairy foods and incident diabetes has been proposed in a new prospective study of the Framingham Heart Study Offspring Cohort [14]. Thus, the contradictory results observed among previous studies may be accounted for by the lack of attention to the baseline glycemic status of participants [14]. The study showed that high-fat dairy and cheese intake had inverse, dose-dependent associations with hazard ratios of incident T2D of 0.70 and 0.63, respectively, in patients with prediabetes when adjusted for baseline glycemia whereas other dairy products, including low-fat dairy, skimmed milk, whole milk, and yogurt, had no association [14]. However, whether dynamic within-person changes over time in the total and subtypes of dairy product consumption are associated with a subsequent risk of T2D among prediabetes patients has not been reported. Evaluating changes in dairy food consumption rather than relying on the baseline intake of individuals is a more robust analytical approach because it can imitate dietary interventions in clinical trials [15]. Thus, in the present analyses, we aim to evaluate the association of changes in total consumption of dairy and subtypes of dairy foods (high-fat milk, low-fat milk, low-fat yogurt, high-fat yogurt, regular cheese, and cream cheese), with subsequent risk of incident T2D. We also used these changes in dairy exposures to estimate the association of replacing specific dairy foods with others on subsequent risk of T2D.

## Method

### Study population

The Tehran Lipid and Glucose Study (TLGS) is a community-based, longitudinal study initiated in 1998 to determine the risk factors for non-communicable diseases in a representative urban population of Tehran. Follow-up visits happened at approximately 3-year intervals. A detailed description of the study design has previously been published [16]. In the fourth examination cycle (2009–2011) of the TLGS, among 12,823 participants who completed questionnaires and a standard medical examination as well as laboratory and anthropometric measurements, a representative sample of participants of 7956 individuals was randomly selected for dietary assessment. Follow-up surveys were conducted in the fifth (2011–2014) and sixth (2015–2018) examinations. We used changes in total dairy consumption and its subtypes from the fourth (2009–2011) to fifth (2011–2014) examination cycles to predict the incidence of T2D risk in the sixth (2015–2018) examination cycle of TLGS. The fourth examination cycle was used as the baseline (initial) for the current analysis.

Among participants, 1401 men and women, aged ≥18 years who had prediabetes (impaired fasting glucose (IFG) [fasting plasma glucose (FPG) levels ≥100 to < 126 mg/dL (≥5.6 to < 7.0 mmol/L), or 2-h plasma glucose (2-hPG) of ≥140 to < 200 mg/dL (≥7.8 to < 11.1 mmol/L) in the fourth examination cycle were selected.

**Fig. 1:**
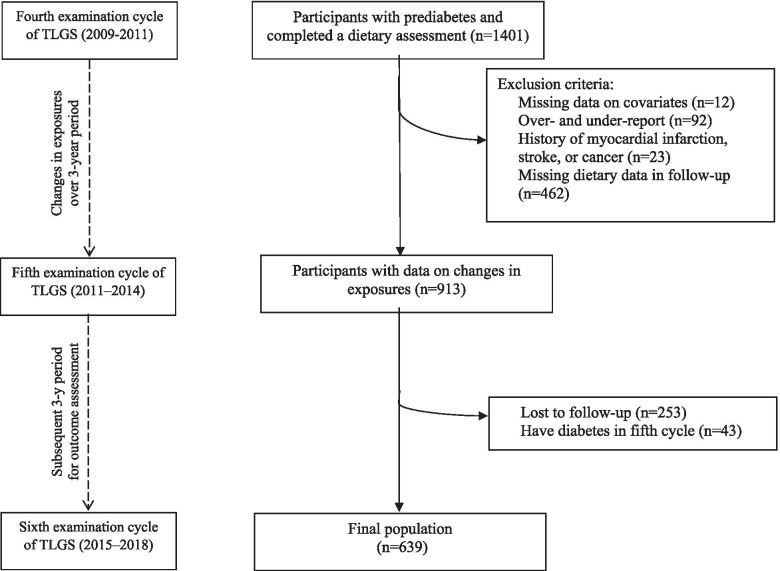
Flow chart of participants included in the present study. TLGS, Tehran lipid and glucose study

Participants were excluded if they reported a history of myocardial infarction, stroke, or cancer at the baseline examination (*n* = 23), if they were missing necessary covariates (*n* = 12), or if they had missing dietary data over follow-up (*n* = 462). We further excluded 92 participants with extreme and implausible energy intake at baseline or in the next examination cycle (defined as < 600 or > 3500 kcal/d for women and < 800 or > 4200 kcal/d for men). Participants were also excluded if they had diagnosed T2D in the fifth cycle (*n* = 43), or they missed the final follow-up examination (*n* = 253). Some participants fell into more than one exclusion category. The final sample size for analysis was 639 participants with prediabetes (Fig. 1). There were no significant differences between participants with follow-up measurements and those lost to follow-up (supplementary Table 1).

All participants signed a written informed consent form. The protocol was approved by the ethics committee of the Research Institute for Endocrine Sciences (IR.SBMU.ENDOCRINE.REC.1399.057), in compliance with the principles of the Declaration of Helsinki.

### Assessment of diet

A semi-quantitative food frequency questionnaire (FFQ) was used to obtain information on the habitual consumption of food items at baseline (fourth cycle) and the fifth examination cycle of TLGS. Trained dietitians determined participants’ intake frequency of food items consumed through the previous year on a daily, weekly, or monthly basis via face-to-face interviews. Portion sizes of consumed foods were reported in household measures, which were then converted to grams.

The exposures of interest in the current analysis, all dairy products, were defined according to the USDA MyPlate (https://www.choosemyplate.gov/dairy) as “foods made from milk that retain their calcium content, including milk, yogurt, cheese, and ice cream.” Total dairy consumption (servings/d) was calculated as the sum of each of the dairy food items in the FFQ, including high-fat milk, low-fat milk, low-fat yogurt, high-fat yogurt, and regular cheese. Low-fat dairy was generated as the sum of low-fat milk plus low-fat yogurt, and high-fat dairy was calculated as the sum of high-fat milk, high-fat yogurt, and regular cheese. One serving of each dairy food item was converted as follows: 240 g for low-fat milk and high-fat milk, 227 g for low-fat yogurt and high-fat yogurt; 28 g for cream cheese and regular cheese; and 120 g for ice cream.

The reproducibility and validity of the FFQ was reported in a previous study [17, 18]. Energy-adjusted correlation coefficients of total dairy consumption based on the FFQ and those based on twelve 24-h dietary recalls among subsamples of men and women were 0.61 and 0.59, respectively. The energy-adjusted intraclass correlation coefficients for the reproducibility of two collected FFQ with a 14-month interval for men and women were 0.48 and 0.66 for total dairy consumption, respectively [18].

### Assessment of outcome and definitions

At baseline and all subsequent examinations, fasting plasma glucose (FPG) was measured in overnight fasting plasma samples with an enzymatic colorimetric method using glucose oxidase (Pars Azmoon, Tehran, Iran). A standard 2-h plasma glucose (2-hPG) test was performed for all participants following oral glucose administration of 82.5 g glucose monohydrate solution [equivalent to 75 g anhydrous glucose; Cerestar EP, Spain]. Participants were classified as having T2D if they reported the use of an oral antidiabetic drug or insulin or had FPG ≥ 126 mg/dL (7 mmol/L) or 2-h PG ≥ 200 mg/dL (11.1 mmol/L).

### Assessment of covariates

Detailed measurements of variables in the TLGS have been reported elsewhere [16]. Briefly, participants were invited to the TLGS center, and trained interviewers collected and updated information at 3-y follow-up intervals regarding the familial history of non-communicable diseases, cigarette smoking, medication usage, and assessment of physical activity. Body height and weight were measured, and BMI was calculated as weight in kg divided by height in meters squared (kg/m^2^). The mean of two measurements of systolic (SBP) and diastolic blood pressure (DBP) after a 15-min rest in the sitting position was recorded as the participant’s blood pressure. Hypertension was defined as an SBP ≥ 140 mmHg or a DBP ≥ 90 mmHg or taking antihypertensive drugs. A valid and reliable Persian version of the Modifiable Activity Questionnaire (MAQ) was applied to measure physical activity and metabolic equivalent (MET) values (MET-min/wk) was calculated.

### Statistical analyses

For total dairy consumption, low-fat, or high-fat dairy products or subtypes of dairy products as the main exposure, categories of change were performed based on the mean and distribution of change of the dairy variables. For instance, individuals were categorized into 3 groups based on changes in total dairy product intake: 1) relatively stable consumption (±0.5 serving/d), 2) increased consumption of > 0.5 serving/d, and 3) decreased consumption of > 0.5 serving/d.

We illustrated the participants’ baseline characteristics and 3-year changes in lifestyle and dietary factors according to the changes in total dairy product consumption using descriptive statistics. The reported means (SD) were adjusted for age, and dietary factors further adjusted for total energy intake. Tests for linear trend across decreasing-stable-increasing categories of changes in total dairy product consumption were performed by assigning the median value of consumption within each category and treating these as continuous variables.

Odds ratio (ORs) and 95% confidence intervals (CIs) across categories of dairy exposures were estimated from multivariable binary logistic regression models by considering incidence of T2D as the dependent variable. Individuals with relatively stable consumption were assigned as the reference group. Model 1 was adjusted for age, sex, physical activity, change in body mass index, family history of diabetes, and total energy intake. Model 2 was further adjusted for dietary factors, including whole-grain intake and energy from protein and carbohydrate. P-trend values were estimated using the median value in each intake category. We further estimated the association of incident of T2D with changes in intakes of dairy products and its subtypes while modeled as continuous variables of 0.5 servings/d.

Lastly, applying cross-product terms, we checked for statistical interaction of changes in total dairy, low- and high- fat dairy with baseline age, sex, and BMI in the final model on the outcome. There were no interactions between those variables and total dairy intake on the incidence of T2D.

We applied substitution modeling to estimate the effect on diabetes risk of increasing the consumption of a subtype of dairy product and simultaneously decreasing consumption of another specific subgroup of dairy product. All type of dairy products were included in the same multivariate model, and OR were calculated from the difference in β coefficients of changes in intakes of different dairy exposure and the 95% CI from the corresponding variances and covariance [19].

We conducted a series of sensitivity analyses to test the robustness of our results: 1) to minimize the influence of outliers, we performed final analysis after excluding participants for whom dairy exposures were lower than the 0.5th percentile or higher than the 99.5th percentile; 2) we additionally adjusted other dietary factors including changes in fruit as well as legumes intakes to measure the impact of these dietary items on the relationship; 3) we further adjusted the final model for 3-year changes in body weight to estimate the extent to which weight change mediates the association between changes in dairy intake with risk of incident T2D; and 4) we added smoking status as a further covariate in final model. All analyses were performed using SPSS software, version 16, and a two-tailed *P* value of 0.05 was considered statistically significant.

## Results

The mean ± SD age of the prediabetes population at baseline was 47.3 ± 11.d y, 50% were women, and 13.8% had a family history of diabetes. We identified 161 cases (25.2%) of incident T2D at the sixth cycle follow-up.

The changes in total, low- and high-fat dairy product consumption were − 0.12 ± 1.45 servings/d, 0.02 ± 1.21 and − 0.16 ± 0.87 serving/d, respectively. Age-adjusted baseline values and changes in physiological markers and lifestyle characteristics according to 3-y changes in total dairy consumption are presented in Table 1. Participants who increased ≥0.5 serving/d total dairy consumption over time had lower initial dairy consumption and total energy intake than those who maintained a stable consumption or decreased their consumption. Baseline percentage of energy from carbohydrate were higher, whereas the percentage of energy from protein, total fat, and SFA slightly decreased among participants who increased total dairy consumption by ≥0.5 serving/d compared with those who maintained a stable consumption. Furthermore, with increasing total dairy intake over time, intake of whole grains (*p* = 0.028) and legumes (*p* = 0.057) tended to be higher. Across all categories of changes in total dairy product consumption, low-fat yogurt and regular cheese were the most consumed dairy products (Supplemental Table 2).

**Table 1:** Adjusted characteristics of participants with prediabetes according to 3-y changes in total dairy consumption

	Changes in total dairy product consumption			P-trend
	Decrease	Relatively stable	Increase	
Initial dairy intake (serving/d)	3.2 (1.1)	2.1 (1.1)	1.8 (1.2)	
Change in dairy intake (serving/d)	−1.44 (0.85)	− 0.02 (0.90)	1.41 (1.00)	
Age (age)	47.4 (11.1)	47.8 (11.8)	46.7 (11.2)	0.552
Female (%)	54.1	50.8	45.8	0.080
Initial body mass index (kg/m^2^)	29.1 (4.9)	29.2 (4.9)	28.7 (4.7)	0.275
Change in body mass index	0.036 (1.4)	0.297 (1.9)	0.223 (1.9)	0.331
Weight change	0.60 (4.03)	1.08 (4.18)	0.83 (4.25)	0.596
Current smoker (%)	5.9	8.8	8.5	0.114
Hypertension (%)	19.8	19.2	19.2	0.832
Family history of diabetes (%)	13.5	10.8	18.1	0.566
Physical activity (MET-min/wk)	429 (54)	394 (51)	403 (60)	0.627
Initial total energy intake (kcal/d)	2203 (710)	2353 (713)	2544 (718)	< 0.001
Change in total energy intake (kcal/d)	340 (1198)	−52 (901)	− 248 (1118)	< 0.001
Carbohydrate (% of total energy)	57.6 (6.4)	58.6 (6.2)	60.4 (6.6)	< 0.001
Fat (% of total energy)	30.6 (6.2)	29.9 (6.2)	28.3 (6.6)	< 0.001
Saturated fatty acids (% of total energy)	10.4 (2.4)	9.3 (2.7)	8.9 (3.4)	< 0.001
Monounsaturated fatty acids (% of total energy)	10.1 (3.0)	9.9 (3.5)	9.6 (2.8)	0.100
Polyunsaturated fatty acids (% of total energy)	5.8 (1.8)	6.1 (1.8)	6.0 (1.9)	0.378
Protein (% of total energy)	15.2 (2.7)	14.9 (2.7)	14.7 (2.6)	0.026
Initial low-fat dairy intake (serving/d)	2.2 (0.8)	1.5 (0.9)	1.3 (0.9)	< 0.001
Change in low-fat dairy intake (serving/d)	−0.80 (0.92)	0.17 (1.00)	0.83 (1.00)	< 0.001
Initial high-fat dairy intake (serving/d)	1.01 (0.89)	0.62 (0.96)	0.53 (0.56)	< 0.001
Change in high-fat dairy intake (serving/d)	−0.53 (0.83)	− 0.19 (0.79)	0.33 (0.79)	< 0.001
Fruit (serving/d)	2.8 (2.1)	2.5 (2.2)	2.6 (2.0)	0.556
Change in fruit (serving/d)	−0.55 (2.45)	0.05 (2.41)	−0.09 (2.42)	0.044
Red and processed meat (serving/d)	0.59 (0.57)	0.66 (0.57)	0.61 (0.57)	0.654
Change in red and processed meat (serving/d)	−0.05 (0.74)	−0.06 (0.73)	0.07 (0.77)	0.199
Whole grains (serving/d)	1.7 (2.6)	2.1 (2.5)	2.3 (2.5)	0.028
Change in whole grains (serving/d)	0.75 (2.66)	1.48 (2.61)	0.48 (2.65)	0.741
Nuts (serving/d)	0.52 (0.78)	0.53 (0.78)	0.56 (0.78)	0.665
Change in nuts (serving/d)	0.06 (1.03)	0.89 (1.02)	0.10 (1.01)	0.870
Legumes (serving/d)	0.42 (0.46)	0.50 (0.40)	0.53 (0.41)	0.057
Change in legumes (serving/d)	0.02 (0.32)	−0.06 (0.35)	−0.02 (0.49)	0.348

Values are mean (SD) or percentage and adjusted for age (except for age). Dietary characteristics are additionally adjusted for energy intake (except for total energy intake)

The risk of incident T2D according to changes in total, low-fat, and high-fat dairy consumption are presented in Table 2. After controlling for age, sex, physical activity, change in body mass index, family history of diabetes, and total energy intake, compared with individuals who maintained a stable consumption (±0.50 serving/d), decreased dairy consumption of ≥0.50 servings/d was associated with 56% higher risk of diabetes at the subsequent 3-year follow-up (OR = 1.56; 95% CI: 1.02 to 2.39). This estimate remained similar after further adjusting for dietary factors, including whole grain intake and percentage of energy from protein and carbohydrate (OR = 1.56; 95% CI: 1.02 to 2.41). Increasing total dairy consumption was not associated with T2D risk compared with maintaining stable consumption. Increasing low-fat dairy consumption by 0.50 serving/d was associated with a 44% (OR = 0.56, 95% CI: 0.35 to 0.90) lower risk of T2D compared with maintaining stable consumption. Null associations between decreasing or increasing consumption of high-fat dairy with the risk of T2D were observed.

**Table 2:** Multivariable adjusted odds ratios (95% confidence intervals) for incident type 2 diabetes according to changes in total, low-fat, and high-fat dairy consumption

	Changes in total dairy product consumption			P for trend
	Decrease	Relatively stable	Increase	
**Total dairy**	> 0.50	±0.50	> 0.50	
Median change	−1.15	− 0.04	1.07	
Case/total	69/222	55/240	37/177	
Age-adjusted OR	1.52 (1.00 to 2.29)	1.00	0.62 (0.55 to 1.42)	0.014
Model 1 OR	1.56 (1.02 to 2.39)	1.00	0.93 (0.58 to 1.51)	0.020
Model 2 OR	1.56 (1.02 to 2.41)	1.00	0.85 (0.52 to 1.39)	0.009
**Low fat dairy**	> 0.50	±0.50	> 0.50	
Median change	−1.05	0.00	1.05	
Case/total	49/172	77/280	35/187	
Age-adjusted OR	1.06 (0.69 to 1.63)	1.00	0.58 (0.37 to 0.92)	0.021
Model 1 OR	1.05 (0.67 to 1.64)	1.00	0.57 (0.36 to 0.91)	0.021
Model 2 OR	1.04 (0.65 to 1.65)	1.00	0.56 (0.35 to 0.90)	0.028
**High fat dairy**	> 0.20	±0.20	> 0.20	
Median change	−0.73	0.00	0.53	
Case/total	63/260	54/213	44/166	
Age-adjusted OR	0.98 (0.64 to 1.50)	1.00	1.20 (0.75 to 1.92)	0.408
Model 1 OR	1.00 (0.64 to 1.57)	1.00	1.35 (0.82 to 2.19)	0.258
Model 2 OR	1.01 (0.62 to 1.58)	1.00	1.36 (0.83 to 2.23)	0.266

Model 1: adjusted for age, sex, physical activity, change in body mass index, family history of diabetes, and total energy intake

Model 2: model 1 plus whole-grain intake and energy from protein and carbohydrate

We further evaluated associations of changes in the consumption of subtypes of dairy products with the risk of T2D (Table 3). Increasing low-fat milk and yogurt consumption was associated with 41% (OR = 0.59, 95% CI: 0.37 to 0.92) and 45% (OR = 0.55, 95% CI 0.33 to 0.93) lower risk of T2D, respectively, than maintaining stable consumption. However, compared with maintaining stable yogurt or low-fat milk consumption, decreasing yogurt and low-fat milk consumption was not associated with T2D risk. Conversely, participants who decreased high-fat milk consumption by ≥0.10 servings/d had a higher risk of T2D (OR = 1.57; 95% CI: 1.02 to 2.41) when compared with individuals who maintained their high-fat milk consumption. Changes in consumption of high-fat yogurt, regular cheese, cream cheese, and ice cream were not associated with T2D risk.

**Table 3:** Multivariable adjusted odds ratios (95% confidence intervals) for incident type 2 diabetes according to changes in subtypes of individual dairy products consumption

	Changes in total dairy product consumption			P for trend
	Decrease	Relatively stable	Increase	
**Low-fat milk**	> 0.10	±0.10	> 0.10	
Median change	−0.32	0.00	0.41	
Case/total	50/140	68/265	43/234	
Age-adjusted	1.52 (0.97 to 2.39)	1.00	0.63 (0.41 to 0.98)	< 0.001
Model 1	1.57 (0.98 to 2.51)	1.00	0.59 (0.38 to 0.93)	< 0.001
Model 2	1.52 (0.95 to 2.45)	1.00	0.59 (0.37 to 0.92)	< 0.001
**High-fat milk**	> 0.10	±0.10	> 0.10	
Median change	−0.41	0.00	0.28	
Case/total	72/244	62/272	27/123	
Age-adjusted	1.48 (0.99 to 2.22)	1.00	1.07 (0.63 to 1.81)	0.106
Model 1	1.49 (0.98 to 2.27)	1.00	1.18 (0.69 to 2.02)	0.204
Model 2	1.57 (1.02 to 2.41)	1.00	1.21 (0.70 to 2.07)	0.163
**High-fat yogurt**	> 0.10	±0.10	> 0.10	
Median change	−0.41	0.00	0.27	
Case/total	38/192	65/253	58/194	
Age-adjusted	0.71 (0.45 to 1.12)	1.00	1.28 (0.84 to 1.96)	0.015
Model 1	0.77 (0.47 to 1.23)	1.00	1.36 (0.87 to 2.11)	0.022
Model 2	0.75 (0.46 to 1.23)	1.00	1.35 (0.86 to 2.10)	0.023
**Low-fat yogurt**	> 0.20	±0.20	> 0.20	
Median change	−0.54	0.00	0.54	
Case/total	59/276	74/274	28/121	
Age-adjusted	1.13 (0.75 to 1.69)	1.00	0.63 (0.39 to 1.05)	0.038
Model 1	1.16 (0.75 to 1.78)	1.00	0.57 (0.34 to 0.96)	0.013
Model 2	1.15 (0.74 to 1.78)	1.00	0.55 (0.33 to 0.93)	0.011
**Regular cheese**	> 0.10	±0.10	> 0.10	
Median change	−0.50	0.00	0.50	
Case/total	37/174	70/268	54/197	
Age-adjusted	0.87 (0.57 to 1.34)	1.00	0.69 (0.42 to 1.12)	0.135
Model 1	0.85 (0.55 to 1.32)	1.00	0.69 (0.42 to 1.15)	0.157
Model 2	0.86 (0.55 to 1.34)	1.00	0.72 (0.43 to 1.18)	0.194
**Cream cheese**	> 0.01	±0.01	> 0.01	
Median change	−0.08	0.00	0.08	
Case/total	70/271	45/166	46/202	
Age-adjusted	1.16 (0.74 to 1.81)	1.00	0.96 (0.62 to 1.49)	0.461
Model 1	1.25 (0.78 to 1.98)	1.00	1.02 (0.65 to 1.60)	0.455
Model 2	1.24 (0.77 to 1.97)	1.00	1.03 (0.65 to 1.63)	0.489
**Ice cream**	> 0.05	±0.05	> 0.05	
Median change	−0.11	0.00	0.11	
Case/total	37/142	94/349	30/148	
Age-adjusted	1.03 (0.66 to 1.62)	1.00	0.74 (0.46 to 1.18)	0.232
Model 1	1.07 (0.67 to 1.73)	1.00	0.75 (0.46 to 1.23)	0.219
Model 2	1.05 (0.65 to 1.69)	1.00	0.77 (0.47 to 1.26)	0.292

Model 1: adjusted for age, sex, physical activity, change in body mass index, family history of diabetes, and total energy intake

Model 2: model 1 plus whole-grain intake, and energy from protein and carbohydrate

**Fig. 2:**
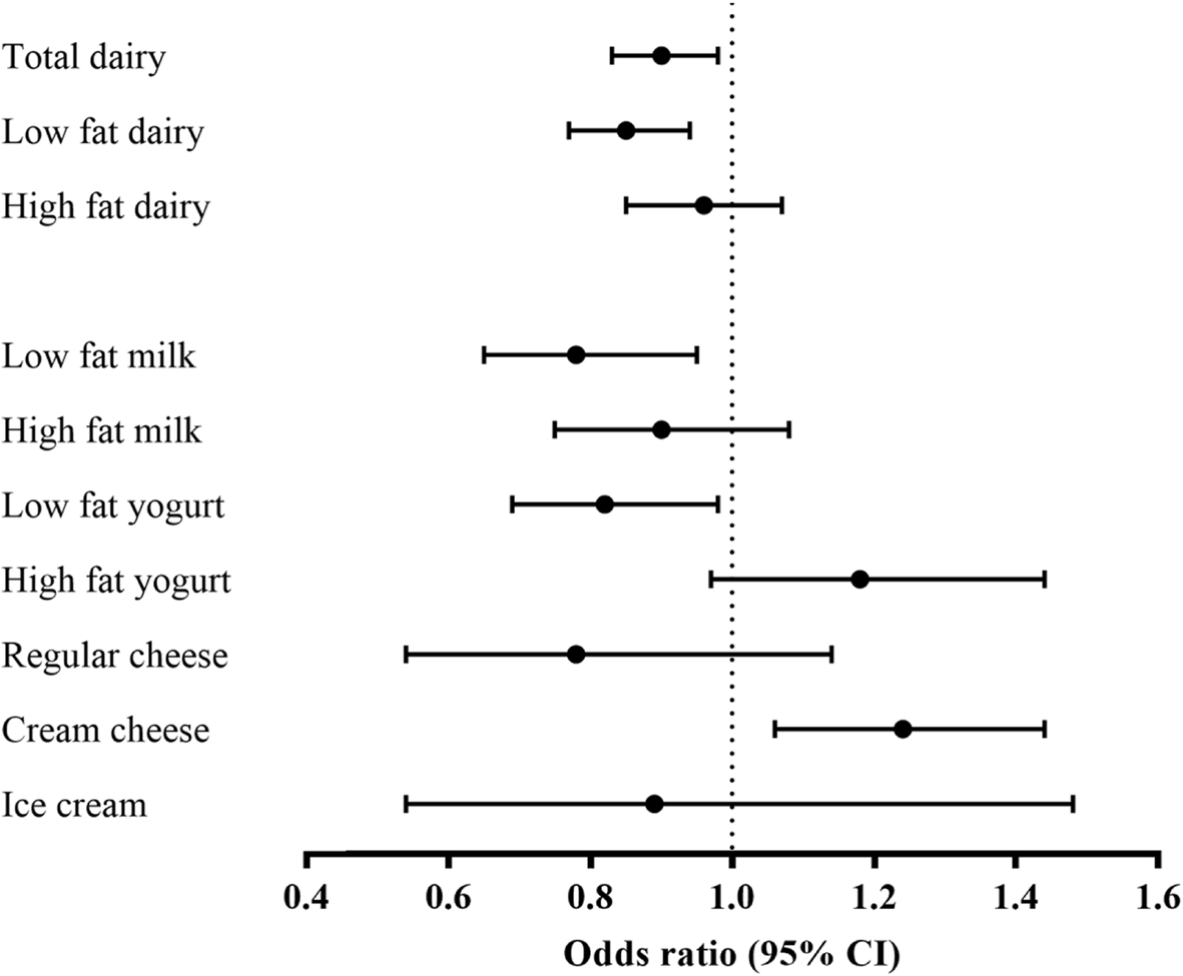
Odds ratios and 95% confidence intervals for incident type 2 diabetes, per 0.5-serving/d increase in consumption of various types of dairy during follow-up. The model was adjusted for age, sex, physical activity, change in body mass index, family history of diabetes, total energy intake, whole-grain intake, and energy from protein and carbohydrate

Each increase of 0.5 servings/d in total dairy and low-fat dairy consumption was associated with an 15% (OR = 0.85; 95% CI: 0.77 to 0.94) and 12% (OR = 0.78; 95% CI: 0.65 to 0.95) lower risk of T2D (Fig. 2). Increasing intakes of low-fat milk and yogurt per 0.5 servings/d were significantly associated with a lower risk of T2D. An increase in consumption of cream cheese (0.5 servings/d) was associated with a higher risk of T2D.

**Fig. 3:**
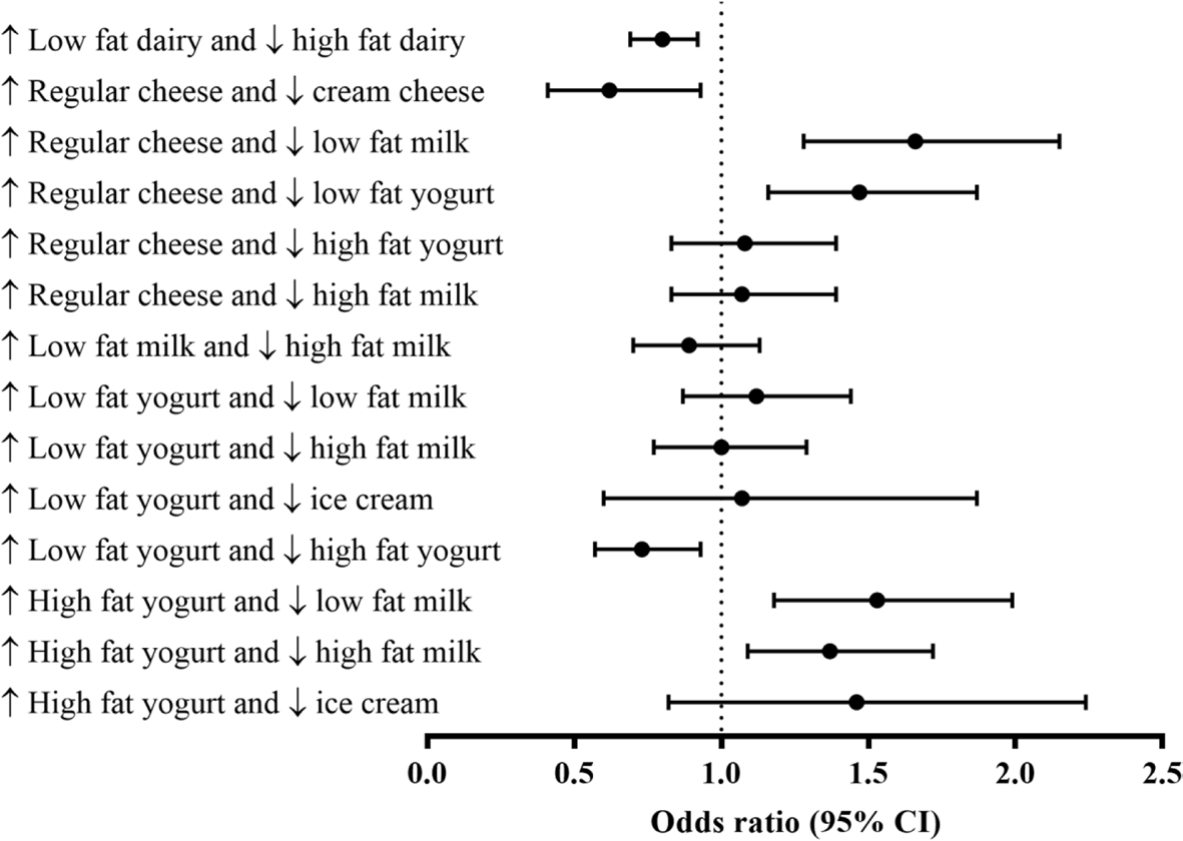
Odds ratios and 95% confidence intervals from substitution models for type 2 diabetes associated with increased consumption of a specific dairy product by 0.5 serving/d and concomitantly decreasing consumption of another dairy product by 0.5 serving/d during a 3-y period. The model was adjusted for age, sex, physical activity, change in body mass index, family history of diabetes, total energy intake, whole-grain intake, and energy from protein and carbohydrate

We estimated that replacing 0.5 daily servings of high-fat dairy with 0.5 daily servings of low-fat dairy was associated with 11% lower diabetes risk in the subsequent 3-year follow-up period (Fig. 3). Increasing low-fat yogurt intake by 0.5 servings/day with a simultaneous decrease in high-fat yogurt was associated with a 27% lower risk of T2D. Replacing low-fat milk and low-fat yogurt by 0.5 servings/d with 0.5 servings/d of regular cheese was associated with 66 and 47% higher risk of T2D, respectively. Substitutions of high-fat yogurt for low-fat milk and high-fat milk were associated with a 53 and 37% higher risk of T2D, respectively, per 0.5 serving/d substituted.

In all sensitivity analyses, excluding subjects with reported outliers, further adjustment for smoking status and concurrent 3-year changes in fruit, legumes intakes, and body weight had small or no impact on the ORs (95%CI) and did not alter the significance of the results (Supplemental Table 3).

## Discussion

In this population-based study of individuals with prediabetes, we observed that increasing low-fat dairy consumption was associated with a 44% lower risk of T2D. Consistent inverse associations with the risk of T2D were observed with a 0.5 increase in the consumption of low-fat dairy. Further, we found that replacing 0.5 daily servings of high-fat dairy with low-fat dairy was associated with an 11% lower risk of diabetes. Also, increased low-fat milk and low-fat yogurt consumption were inversely associated with incident T2D. Thus, these findings suggest a potential role of the fat content of dairy foods concerning the development of T2D. This study benefits from a longitudinal design to imitate a large nutritional intervention investigating an aspect of changes in dairy products through a regular diet when individuals were living with prediabetes and in the diabetes development over a long-time period.

Compared with participants who had stable total dairy consumption, participants who decreased their total dairy intake had a 56% higher risk of subsequent incidence of T2D. Each 0.5 serving/d increased in total dairy consumption over 3-year was also associated with a 15% lower risk of subsequent diabetes. In a very recent study of 3 US cohorts of healthy participants, among those with normoglycemia at baseline, participants who decreased their total dairy consumption more than 1 serving/d through a 4-y period had 11% increased risk of T2D, compared with those who had a relatively stable intake [20]. Reducing the intake of total dairy products over time may increase the progression of T2D because dairy foods are being replaced by other, less healthy foods. For instance, those who decrease their dairy intakes may increase the consumption of sugar-sweetened beverages, which could result in a higher risk of T2D.

Although there are other studies supporting a favorable association of total dairy intake with risk of T2D [21–23], it seems that some inherent properties of dairy foods may influence their effects on T2D risk, including the type and fat content of the dairy products. Their overall benefit in specific populations may also relate to the general popularity in their consumption, which may differ according to ethnocultural considerations [24]. Regarding the fat content of dairy products, in accordance with previous studies associating low-fat dairy consumption with T2D incidence among apparently healthy individuals [23], we observed that long-term increases in low-fat dairy intake were associated with subsequent lower T2D risk in people with prediabetes. A meta-analysis done by pooling data from observational studies showed that both low-fat and high-fat dairy consumption had no association with the risk of T2D [25]. Although our finding showed a non-significant association between increased high-fat dairy consumption over time and subsequent risk of T2D, results from the Framingham Heart Study Offspring Cohort illustrated that higher consumption of high-fat dairy decreased the risk of incident T2D among prediabetes participants by 70% [14]. Current data from the international cohort of The Prospective Urban Rural Epidemiology (PURE) study has shown that higher consumption of whole-fat (but not low-fat) dairy is associated with a lower risk of T2D compared with no dairy intake [26]. Consistent with our findings, a US cohort study revealed that compared with those stable in their intake, participants who decreased their low-fat dairy intake by > 0.5 daily serving had a higher subsequent risk of T2D, and no significant relationship between high-fat dairy and diabetes was observed [20]. We also estimated that substituting low-fat dairy for high-fat dairy products was favorably associated with the incidence of T2D. Findings from previous study [20] and results from ours highlight the preventive association of low-fat dairy on T2D.

In our analysis, increasing low-fat milk consumption was associated with lower T2D risk relative to the reference category. Furthermore, an increase of 0.5 servings/d in low-fat milk consumption was inversely associated with the risk for T2D. However, there was no significant relationship between high-fat milk and the risk of T2D. Evidence for an association of milk consumption with the risk of T2D is inconsistent. The meta-analysis by Gijsbers et al. [12] indicated that total milk consumption was not associated with T2D risk. Null associations of the total, low- and high-fat milk consumption with incident T2D were also demonstrated in a study of participants with prediabetes [14]. However, a protective association of higher intake of low-fat milk was reported in some studies [27, 28]. A study investigating the association of changes in low-fat milk consumption with the incidence of T2D among normoglycemic subjects indicated that decreasing consumption of low-fat milk was associated with a higher risk of T2D [20].

Fermented dairy products, including yogurt and cheese, may elicit different impacts on the metabolic status of individuals than non-fermented dairy products. Increasing low-fat yogurt over 3 years decreased the risk of T2D by 55% compared with those with relatively stable intake, whereas decreasing high-fat yogurt intake was also associated with benefits. Yogurt consumption, regardless of fat content, was associated with a lower risk of T2D expressed in meta-analyses of prospective cohort studies [12, 28]. A nonlinear association for yogurt consumption was observed with incident T2D among participants with prediabetes, and no additional protective relation was observed with greater than 1–3 servings/week [14]. It was recently illustrated in a pooled analysis of 3 US cohorts that participants decreasing yogurt consumption by > 1.0 serving/d over a 4-y period had an 11% higher risk of T2D when compared to those with stable yogurt consumption [20]. On the other hand, increasing cream cheese consumption over 3 years increased the risk of subsequent T2D in our population; however, increasing the consumption of regular cheeses was not associated with T2D risk. Regarding the association of cheese consumption and T2D, positive [20], inverse [14], and no [29] associations were reported in different cohort studies. These contradictory results between different observational studies might be accounted for by a lack of ability to differentiate among the various types of cheeses, which contain different amounts of fat and sodium. It should be noted that most cheeses, even those with low fat content, contain relatively high amounts of fat, which may increase the amount of SFAs in the diet.

Since increasing consumption over time of low-fat dairy products, including low-fat milk and low-fat yogurt, was associated with a lower risk of T2D, these results highlight the potentially favorable effect of moderate amounts of dairy fat intake on T2D. In this regard, when regular cheese (high-fat content) replaced 0.5 servings/d of low-fat milk or low-fat yogurt over the 3-year period, the the subsequent risk of diabetes increased by 66 and 47%, respectively. Similarly, replacing high-fat yogurt with low-fat milk or low-fat yogurt was associated with lower T2D risk. It should be noted that, except for cream cheese, all substitutions in other types of high-fat dairy had no association with the onset of T2D.

Physiologically, the association between an increase in low-fat dairy and a lower increase in body weight and BMI over time [30] may at least partially account for its association with reduced incident T2D. From a practical standpoint, replacing one type of dairy product for another may be more feasible than substituting for other food groups in a real-life setting. Thus, substituting high-fat dairy products such as cheese with low-fat dairy products such as low-fat milk or low-fat yogurt may be acceptable for patients with prediabetes to prevent the onset of diabetes. However, as dairy fat contains a complex mix of different SFAs with different biological effects and given the pre-existing metabolic states on differential response to the foods [31], further mechanistic studies and prospective studies with substitution models among participants with impaired glucose tolerance are needed to get a complete picture of dairy consumption and T2D risk.

Strengths of this study include that the TLGS cohort is population-based with reasonable representation of the urban population of Tehran [16], and the diagnosis of prediabetes and diabetes was defined based on a drawn fasting blood sample. In addition, we examined the temporality of exposures and outcomes. The FFQ was collected through a careful interview utilizing a photographic method to minimize the impact of recall bias. Furthermore, in this study, baseline characteristics of participants lost to follow-up compared to those followed did not have meaningful differences; thus selection bias is unlikely to have influenced the results.

Several limitations of this investigation need to be mentioned. First, using an FFQ cannot estimate the ‘actual’ intake of participants. For example, cheese is commonly consumed in mixed meals and prepared foods. Second, despite controlling for different covariates in our analysis, residual confounding due to unknown or unmeasured confounders cannot be excluded. Finally, this study was conducted on data from TLGS, which is a homogeneous urban population; thus, results may not be generalizable to other populations, such as rural dwelling or other those with other ethnocultural backgrounds.

## Conclusion

In conclusion, in this prospective study of participants with prediabetes, decreasing total dairy consumption was positively associated with incident T2D. Increasing low-fat dairy consumption or replacing high-fat dairy with low-fat products was generally associated with a lower risk of diabetes. Our analysis also suggests that substituting regular cheese for low-fat milk or low-fat yogurt increased the risk of T2D in people with prediabetes.

## Supplementary Information

**Additional file 1:** **Supplementary Table 1.** A comparison of baseline characteristics between participants followed and those lost to follow-up.

**Additional file 2:** **Supplementary Table 2.** Initial intake and changes in consumption of subtypes of dairy products over a 3-year follow-up.

**Additional file 3:** **Supplementary Table 3.** Sensitivity analysis: Multivariable adjusted odds ratios (95% confidence intervals) for incident type 2 diabetes according to categories changes in total, low-fat, and high-fat dairy consumption.

## Data Availability

The data set is the property of Research Institute for Endocrine Sciences (RIES) and is made available upon approval of the research proposal by the research council and the ethics committee. The RIES ethics committee must issue an approval in case of a request for access to the de-identified dataset. Data request may be sent to the head of the RIES Ethics Committee Dr. Azita Zadeh-Vakili at email: azitavakili@endocrine.ac.ir.

## Abbreviations

FBS: Fasting plasma glucose
T2D: Type 2 diabetes
TLGS: Tehran Lipid and Glucose Study
OR: Odds ratio
IFG: Impaired fasting glucose
2-hPG: 2-h plasma glucose
FFQ: Food frequency questionnaire
