# Correction to: Changes in dairy product consumption and subsequent type 2 diabetes among individuals with prediabetes: Tehran lipid and glucose study

**DOI:** 10.1186/s12937-021-00754-w

**Published:** 2021-12-22

**Authors:** Emad Yuzbashian, Golaleh Asghari, Parvin Mirmiran, Catherine B. Chan, Fereidoun Azizi

**Affiliations:** 1 Nutrition and Endocrine Research Center, Research Institute for Endocrine Sciences, Shahid Beheshti University of Medical Sciences, Tehran, Iran; 2 Department of Agricultural, Food and Nutritional Science, University of Alberta, Edmonton, Alberta Canada; 3 Department of Clinical Nutrition and Dietetics, Faculty of Nutrition Sciences and Food Technology, National Nutrition and Food Technology Research Institute, Shahid Beheshti University of Medical Sciences, Tehran, Iran; 4 Department of Physiology, University of Alberta, Edmonton, Alberta Canada; 5 Endocrine Research Center, Research Institute for Endocrine Sciences, Shahid Beheshti University of Medical Sciences, Tehran, IR Iran

**Correction to: Nutr J 20, 88 (2021).**

**https://doi.org/10.1186/s12937-021-00745-x**

Following publication of the original article [1], it came to the authors’ attention that affiliations 1 and 2 had been erroneously swapped.

Namely, ‘Department of Agricultural, Food and Nutritional Science, University of Alberta, Edmonton, Alberta, Canada’ was written in place of ‘Nutrition and Endocrine Research Center, Research Institute for Endocrine Sciences, Shahid Beheshti University of Medical Sciences, Tehran, Iran’, and vice versa.

The error has since been corrected in the original article and the corrected affiliation information may be seen in this correction.

The authors apologize for any inconvenience caused.
