# Supplementary material for: Changes in dairy product consumption and subsequent type 2 diabetes among individuals with prediabetes: Tehran Lipid and Glucose Study
Source: Nutr J. 2021 Oct 29;20:88. doi: 10.1186/s12937-021-00745-x (PMC8556890; doi:10.1186/s12937-021-00745-x)
Supplement: Supplementary file 1 — Additional file 1: Supplementary Table 1. A comparison of baseline characteristics between participants followed and those lost to follow-up. [file 12937_2021_745_MOESM1_ESM.docx]

Supplementary Table 1- A comparison of baseline characteristics between participants followed and those lost to follow-up

|  | With follow-up measurements (n=639) | Lost to follow-up (n=253) | P-value |
| --- | --- | --- | --- |
| Age (year) | 48.1 (12.5) | 47.3 (15.6) | 0.445 |
| Female (%) | 48.5 | 43.7 | 0.145 |
| Body mass index (kg/m^2^) | 29.1 (4.6) | 29.1 (4.8) | 0.979 |
| Total energy intake (kcal/d) | 2622 (1160) | 2573 (1108) | 0.507 |
| Fasting plasma glucose (mg/dl) | 104.7 (10.2) | 104.7 (9.4) | 0.942 |
| Physical activity (MET-min/wk) | 434.5 (847.8) | 416.6 (767.5) | 0.741 |

Data are presented as mean (SD) or percentage

Two-tailed T-test was used and p=0.05 considered significant
