# Supplementary material for: Changes in dairy product consumption and subsequent type 2 diabetes among individuals with prediabetes: Tehran Lipid and Glucose Study
Source: Nutr J. 2021 Oct 29;20:88. doi: 10.1186/s12937-021-00745-x (PMC8556890; doi:10.1186/s12937-021-00745-x)
Supplement: Supplementary file 2 — Additional file 2: Supplementary Table 2. Initial intake and changes in consumption of subtypes of dairy products over a 3-year follow-up. [file 12937_2021_745_MOESM2_ESM.docx]

Supplementary Table 2- Initial intake and changes in consumption of subtypes of dairy products over a 3-year follow-up.

|  | Changes in total dairy product consumption | | |
| --- | --- | --- | --- |
|  | Decrease | Relatively stable | Increase |
| Low-fat milk (serving/d) |  |  |  |
| Initial | 0.26 (0.38) | 0.19 (0.38) | 0.18 (0.38) |
| Change | -0.05 (0.53) | 0.14 (0.52) | 0.23 (0.53) |
| High-fat milk (serving/d) |  |  |  |
| Initial | 0.51 (0.48) | 0.29 (0.48) | 0.37 (0.47) |
| Change | -0.34 (0.50) | -0.15 (0.49) | 0.70 (0.49) |
| Low-fat yogurt(serving/d) |  |  |  |
| Initial | 0.69 (0.47) | 0.52 (0.48) | 0.45 (0.47) |
| Change | -0.33 (0.57) | -0.045 (0.57) | 0.21 (0.57) |
| High-fat yogurt(serving/d) |  |  |  |
| Initial | 0.36 (0.42) | 0.28 (0.58) | 0.24 (0.43) |
| Change | -0.16 (0.59) | -0.05 (0.57) | 0.22 (0.58) |
| Regular cheese(serving/d) |  |  |  |
| Initial | 0.91 (0.59) | 0.68 (0.58) | 0.60 (0.58) |
| Change | -0.32 (0.66) | 0.06 (0.66) | 0.35 (0.66) |
| Cream cheese(serving/d) |  |  |  |
| Initial | 0.07 (0.19) | 0.06 (0.18) | 0.10 (0.18) |
| Change | 0.00 (0.26) | 0.02 (0.26) | 0.02 (0.26) |
| Ice cream(serving/d) |  |  |  |
| Initial | 0.10 (0.13) | 0.07 (0.12) | 0.08 (0.13) |
| Change | -0.03 (0.19) | 0.01 (0.18) | 0.06 (0.18) |

Values are mean (SD), which are adjusted for baseline age and energy intake.
