# Supplementary material for: Changes in dairy product consumption and subsequent type 2 diabetes among individuals with prediabetes: Tehran Lipid and Glucose Study
Source: Nutr J. 2021 Oct 29;20:88. doi: 10.1186/s12937-021-00745-x (PMC8556890; doi:10.1186/s12937-021-00745-x)
Supplement: Supplementary file 3 — Additional file 3: Supplementary Table 3. Sensitivity analysis: Multivariable adjusted odds ratios (95% confidence intervals) for incident type 2 diabetes according to categories changes in total, low-fat, and high-fat dairy consumption. [file 12937_2021_745_MOESM3_ESM.docx]

Supplementary table 3- Sensitivity analysis: Multivariable adjusted odds ratios (95% confidence intervals) for incident type 2 diabetes according to categories changes in total, low-fat, and high-fat dairy consumption

|  | Changes in total dairy consumption | | | P for trend |
| --- | --- | --- | --- | --- |
|  | Decrease | Relatively stable | Increase |  |
| *Sensitivity Analysis (1)* |  |  |  |  |
| **Total dairy^1^** | >0.50 | ±0.50 | >0.50 |  |
| Case/total | 69/219 | 55/240 | 36/173 |  |
| Model 1 | 1.62 (1.07 to 2.49) | 1.00 | 0.91 (0.56 to 1.49) | 0.014 |
| **Low-fat dairy^1^** | >0.50 | ±0.50 | >0.50 |  |
| Case/total | 49/172 | 77/280 | 35/187 |  |
| Model 1 | 1.00 (0.64 to 1.56) | 1.00 | 0.55 (0.34 to 0.87) | 0.021 |
| **High-fat dairy^1^** | >0.20 | ±0.20 | >0.20 |  |
| Case/total | 63/260 | 54/213 | 44/166 |  |
| Model 2 | 0.96 (0.62 to 1.500) | 1.00 | 1.23 (0.76 to 2.00) | 0.321 |
| *Sensitivity Analysis (2)* |  |  |  |  |
| **Total dairy** | >0.50 | ±0.50 | >0.50 |  |
| Case/total | 69/225 | 55/241 | 37/173 |  |
| Model 2 | 1.63 (1.05 to 2.52) | 1.00 | 0.92 (0.57 to 1.50) | 0.018 |
| **Low-fat dairy** | >0.50 | ±0.50 | >0.50 |  |
| Case/total | 49/172 | 77/280 | 35/187 |  |
| Model 2 | 0.96 (0.61 to 1.50) | 1.00 | 0.57 (0.35 to 0.90) | 0.041 |
| **High-fat dairy** | >0.20 | ±0.20 | >0.20 |  |
| Case/total | 63/260 | 54/213 | 44/166 |  |
| Model 2 | 0.94 (0.61 to 1.47) | 1.00 | 1.23 (0.76 to 1.98) | 0.284 |
| *Sensitivity Analysis (3)* |  |  |  |  |
| **Total dairy** | >0.50 | ±0.50 | >0.50 |  |
| Case/total | 69/225 | 55/241 | 37/173 |  |
| Model 3 | 1.64 (1.07 to 2.53) | 1.00 | 0.93 (0.57 to 1.52) | 0.018 |
| **Low-fat dairy** | >0.50 | ±0.50 | >0.50 |  |
| Case/total | 49/172 | 77/280 | 35/187 |  |
| Model 3 | 0.99 (0.63 to 1.54) | 1.00 | 0.56 (0.35 to 0.89) | 0.041 |
| **High-fat dairy** | >0.20 | ±0.20 | >0.20 |  |
| Case/total | 63/260 | 54/213 | 44/166 |  |
| Model 3 | 0.96 (0.61 to 1.49) | 1.00 | 1.25 (0.77 to 2.02) | 0.321 |
| *Sensitivity Analysis (4)* |  |  |  |  |
| **Total dairy** | >0.50 | ±0.50 | >0.50 |  |
| Case/total | 69/222 | 55/240 | 37/177 |  |
| Model 4 | 1.58 (1.03 to 2.44) | 1.00 | 0.94 (0.58 to 1.53) | 0.022 |
| **Low-fat dairy** | >0.50 | ±0.50 | >0.50 |  |
| Case/total | 49/172 | 77/280 | 35/187 |  |
| Model 4 | 0.99 (0.64 to 1.56) | 1.00 | 0.57 (0.36 to 0.90) | 0.033 |
| **High-fat dairy** | >0.20 | ±0.20 | >0.20 |  |
| Case/total | 63/260 | 54/213 | 44/166 |  |
| Model 4 | 0.97 (0.62 to 1.51) | 1.00 | 1.27 (0.78 to 1.51) | 0.281 |

^1^We performed analysis on participants with values between 0.5^th^ percentile and the 99.5^th^ of corresponding exposures

Model 1: adjusted for age, sex, physical activity, change in body mass index, family history of diabetes, and total energy intake, initial whole grain intake, and energy from protein and carbohydrate

Model 2: adjusted for age, sex, physical activity, change in body mass index, family history of diabetes, and total energy intake, initial legumes, whole grain and changes in fruit intake, and energy from protein and carbohydrate

Model 3: adjusted for age, sex, physical activity, change in body mass index, family history of diabetes, and total energy intake, initial whole grain, energy from protein and carbohydrate, and weight change.

Model 4: adjusted for age, sex, physical activity, change in body mass index, family history of diabetes, smoking status, and total energy intake, initial whole grain, and energy from protein and carbohydrate.
